# Association between skeletal muscle and left ventricular mass in patients with hyperthyroidism

**DOI:** 10.3389/fendo.2024.1301529

**Published:** 2024-01-31

**Authors:** Zhenchao Liu, Guang Liu, Yanzhi Wang, Chongwen Zheng, Yunliang Guo

**Affiliations:** ^1^ Institute of Integrative Medicine, Qingdao University, Qingdao, Shandong, China; ^2^ Shandong Provincial Sports Center, Shandong Administration of Sports, Jinan, Shandong, China; ^3^ Academic Affairs Office, Binzhou Medical University, Yantai, Shandong, China; ^4^ Department of Neurology, The 2nd Affiliated Hospital of Fujian University of Traditional Chinese Medicine, Fuzhou, Fujian, China

**Keywords:** hyperthyroidism, sarcopenia, muscle atrophy, left ventricular mass, cardiac remodeling

## Abstract

**Objective:**

This study aims to investigate the relationship between skeletal muscle and left ventricular mass (LVM) in patients with hyperthyroidism, providing theoretical and data-based foundations for further research on the interaction between secondary muscle atrophy and cardiac remodeling.

**Methods:**

A retrospective data collection was conducted, including 136 patients with hyperthyroidism (Study group) and 50 healthy participants (control group). The Study group was further divided into Group A (high LVM) and Group B (low LVM) based on LVM size. Multiple linear regression analysis was performed to examine the correlation between skeletal muscle and LVM, with model evaluation. Based on the results, further nonlinear regression analysis was conducted to explore the detailed relationship between skeletal muscle and LVM.

**Results:**

Compared to the control group, the Study group exhibited significantly lower LVM, skeletal muscle mass index (SMI), and skeletal muscle mass (SMM) (*P*<0.05). Within the subgroups, Group A had significantly higher SMI, SMM, and hand grip strength compared to Group B (*P*<0.05). The results of the multiple linear regression showed a certain correlation between SMI (*β*=0.60, *P*=0.042, *95% CI*=0.02~1.17) and hand grip strength (*β*=0.34, *P*=0.045, *95% CI*=0.01~0.67) with LVM. However, the residuals of the multiple regression did not follow a normal distribution (K-S=2.50, P<0.01). Further results from a generalized linear model and structural equation modeling regression also demonstrated a correlation between SMI (*β*=0.60, *P*=0.040, *95% CI*=0.03~1.17) (*β*=0.60, *P*=0.042, *95% CI*=0.02~1.17) and hand grip strength (*β*=0.34, *P*=0.043, *95% CI*=0.01~0.67) (*β*=0.34, *P*=0.045, *95% CI*=0.01~0.67) with LVM.

**Conclusion:**

Patients with hyperthyroidism may exhibit simultaneous decreases in LVM, SMM, and SMI. The LVM in patients is correlated with SMM and hand grip strength, highlighting the need for further exploration of the causal relationship and underlying mechanisms. These findings provide a basis for the prevention and treatment of secondary sarcopenia and cardiac pathology in patients with hyperthyroidism.

## 1 Background

Sarcopenia is a condition characterized by the loss of skeletal muscle mass and decline in muscle strength, it can result in reduced physical activity capacity, increased risk of falls, fractures, and disability. The main causes of sarcopenia include age-related imbalances in nutrition intake, chronic inflammation, endocrine and metabolic disorders, as well as secondary skeletal muscle loss caused by chronic wasting diseases (1, 2). Previous studies have primarily focused on skeletal muscle atrophy, but recent research suggests that smooth muscle and cardiac muscle also exhibit muscle atrophy (3, 4). During the aging process, the heart undergoes changes characterized by left ventricular hypertrophy and increased mass. These changes are typically attributed to elevated systolic pressure or inadequate blood supply caused by arterial stiffness. Existing research has primarily focused on myocardial cell hypertrophy, with limited investigation into myocardial cell loss, which is considered to be a preceding phenomenon before adaptive hypertrophy. Understanding the relationship between skeletal muscle and cardiac muscle during the aging process is of significant importance for understanding the upstream pathological changes preceding the occurrence of clinical cardiovascular diseases and the terminal changes in cardiovascular prognosis (4, 5). Several studies have shown a potential correlation between parameters such as skeletal muscle mass and left ventricular mass (LVM). Keng et al. (4) found that in the elderly non-cardiac population, both skeletal muscle and cardiac muscle exhibited a decrease in mass with aging, proposing the concept of “Cardio-Sarcopenia.” Patients with sarcopenia have lower LVM and diameter, and hand grip strength is linearly correlated with LVM. Pelà et al. (6) found a significant positive correlation between limb lean mass and LVM in frail and sarcopenic patients aged 70 and above, further confirming the coexistence of skeletal muscle and cardiac muscle weakness in the elderly population. Tinti et al. (7) observed a significant correlation between LVM and lean body mass, limb skeletal muscle mass, skeletal muscle mass index (SMI), and total body bone mass in subjects aged 65 to 91 years old. These studies have primarily focused on the elderly population aged 65 and above, with participants experiencing sarcopenia due to aging. However, research on secondary sarcopenia is relatively limited. Patients with hyperthyroidism, for example, experience muscle mass loss as elevated thyroid hormone levels can increase muscle breakdown, leading to muscle wasting and weakness (8, 9). Unlike the elderly population, patients with hyperthyroidism are often middle-aged or younger, making it necessary to study the interaction between skeletal muscle and LVM in this population. The aim of this study is to investigate the changes in skeletal muscle and LVM in patients with hyperthyroidism and understand the potential interactions between skeletal muscle and LVM in this group.

## 2 Participants and methods

### 2.1 Participants

**Figure 1:**
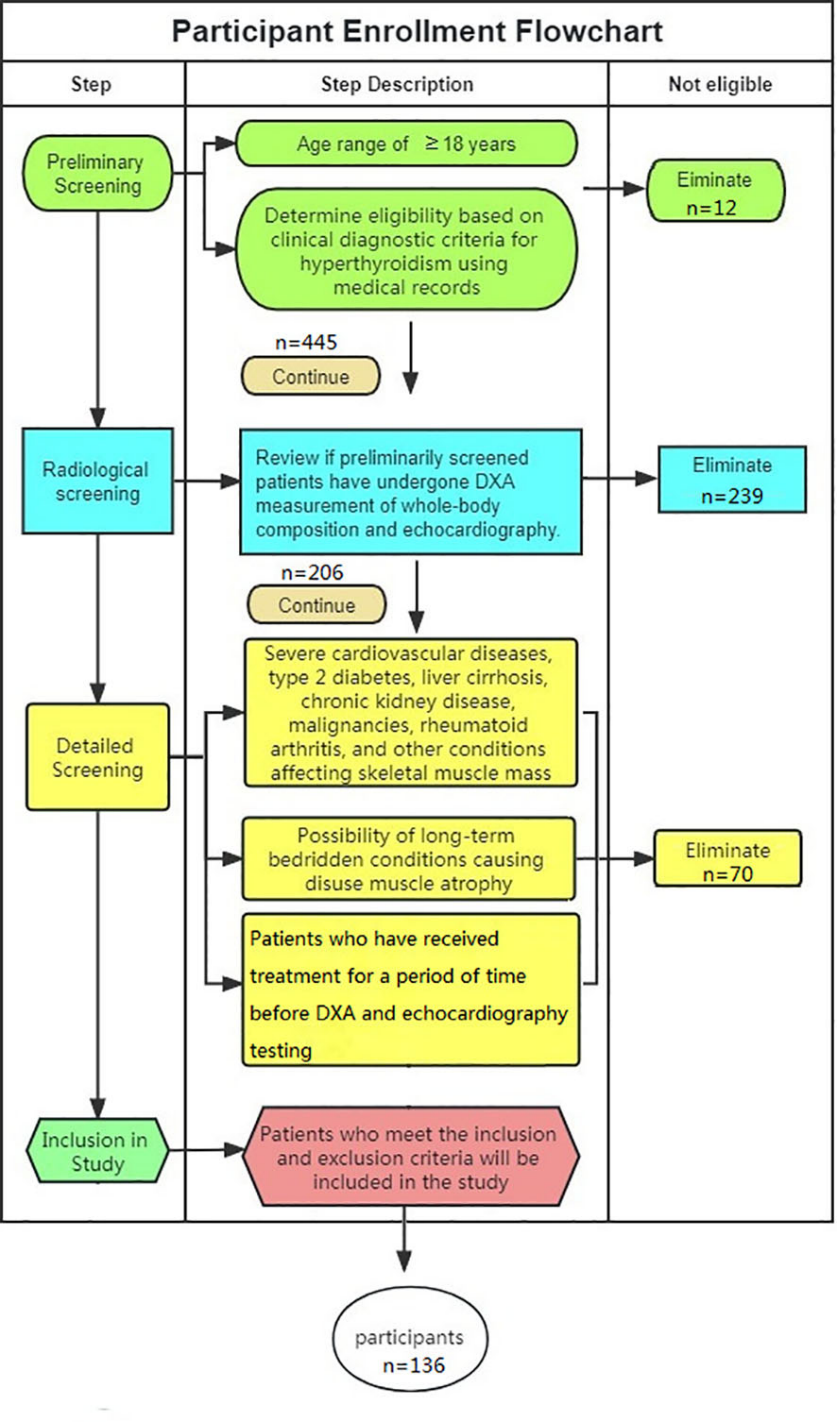
Flowchart of participant with hyperthyroidism inclusion process.

A retrospective analysis was conducted on patients with hyperthyroidism who underwent dual-energy X-ray absorptiometry (DXA) for skeletal muscle assessment at the 11th Clinical College of Qingdao University from January 2017 to January 2021. Additionally, 50 randomly selected individuals undergoing health check-ups were included as the control group. Following the inclusion and exclusion criteria, a total of 186 participants were included in the study, with 136 in the Study group and 50 in the control group (Participants with hyperthyroidism were screened as shown in **Figure 1**). The Study group was further divided into two subgroups, Group A (participants with high left ventricular mass, LVM) and Group B (participants with low LVM). This study was approved by the Medical Ethics Committee of the 11th Clinical College of Qingdao University (Ethics Review Number: YX30068) and conducted in accordance with the principles outlined in the Helsinki Declaration.

Diagnostic Criteria: The diagnostic criteria for sarcopenia and pre-sarcopenia were based on the Asian Working Group for Sarcopenia 2019 diagnostic criteria (10). Participants were considered to have muscle reduction if their dual-energy X-ray examination showed skeletal muscle mass index (SMI) <7.0 kg/m^2^ for males and SMI <5.4 kg/m^2^ for females; hand grip strength <28 kg for males and <18 kg for females.

Inclusion Criteria: (1) Age ≥18 years old; (2) Participants who underwent echocardiography examination; (3) Participants who underwent hand grip strength testing; (4) Hyperthyroidism patients who underwent serum Interleukin-6 (IL-6), Interleukin-15 (IL-15), and Tumor necrosis factor-alpha (TNF-α) testing; (5) Hyperthyroidism patients with complete clinical data including gender, age, medical history, lipid profile, blood pressure, thyroid hormones, and weight.

Exclusion Criteria: (1) Participants with pre-existing severe cardiovascular diseases leading to structural changes in the heart prior to hyperthyroidism; (2) Participants with diseases such as type 2 diabetes, liver cirrhosis, chronic renal insufficiency, malignant tumors, rheumatoid arthritis, and other conditions that can affect skeletal muscle mass; (3) Participants with a possibility of prolonged immobilization and disuse muscle atrophy due to surgical fractures or other reasons; (4) Considering the impact of medication treatment on muscles, patients who have received treatment for a period of time before DXA and echocardiography testing are not included.

### 2.2 Research methods

#### 2.2.1 Data collection

The aim of this study was to investigate retrospectively the association between skeletal muscle parameters and LVM in patients with hyperthyroidism. Clinical data of all eligible participants were collected as covariates, including age, gender, height, weight, smoking status (Smokers: Every day smoking more than 20 cigarettes, continuously smoking for more than 10 years, or smoking more than 40 cigarettes every day, continuously smoking for more than 5 years), drinking status (Drinkers: Drinking alcohol for more than 300 days per year or consuming more than 120 gr of alcohol per month), lipid profile, blood pressure, medical history, Hand grip (WCS-10000, Wanqing, China), Free Triiodothyronine (FT3), Free Triiodothyronine (FT4), Thyroid-Stimulating Hormone (TSH), IL-6 (kit No. SUB10377, Shunbo, China), IL-15 (kit No. SUB11237, Shunbo, China) and TNF-α (kit No. SUB11776, Shunbo, China) levels. Body mass index (BMI) = weight (kg)/height (m^2^).

Based on DXA (GE, USA) scan results, total skeletal muscle mass (SMM), upper-limb skeletal muscle mass (USMM), and lower-limb skeletal muscle mass (LSMM) were collected for all participants. Skeletal muscle index (SMI) = appendicular lean mass (ALM) (kg)/height (m^2^).

Furthermore, Echocardiography (EPIQ7, PHILIPS, Netherlands) was performed to collect data on left atrium (LA), aorta (AO), left ventricular end-diastolic diameter (LVDd), interventricular septal thickness at end-diastole (IVSTd), left ventricular posterior wall thickness at end-diastole (LVPWTd), right ventricle (RV), ascending aorta (AAO), main pulmonary artery (MPA), ejection fraction (EF), and fractional shortening (FS). Left ventricular mass (LVM) was calculated using the formula LVM = 0.8×1.04×[(LVDd + LVPWTd + IVSTd) 3-LVDd3] + 0.6.

#### 2.2.2 Statistical processing

Comparisons between the Study group and the control group, as well as between subgroups, were conducted using t-tests or Mann-Whitney U tests for continuous data comparisons, and chi-square tests for categorical data comparisons. To explore the correlation between skeletal muscle and LVM, this study initially performed multiple linear regression analysis and further examined the normality of residuals. If the residuals did not follow a normal distribution assumption, further nonlinear regression analysis was conducted. Nonlinear regression was performed using the locally weighted scatterplot smoothing (LOWESS) method, generalized linear models (GLM), and structural equation models (SEM). Regularization was applied to stabilize parameter estimation and test model stability. The statistical analysis and model development were conducted using Python 3.10 (Python Software Foundation, https://www.python.org). All statistical results were considered statistically significant at a significance level of *P*<0.05.

## 3 Results

### 3.1 Participant characteristics

#### 3.1.1 Comparison of variables between the two groups

Compared to the control group, the Study group had lower SMI, SMM, and LVM (*P*<0.05), while there was no significant difference in grip strength between the two groups (*P*>0.05) (see **Table 1**).

**Table 1:** The comparison of variables between the study group and the control group.

Variable	Study group (n=136)	Control group (n=50)	*t*/*z*/*x* ^2^	*P*	95%CI
Clinical covariates					
Age (years)(*x̄* ± *s*)	50.79 ± 16.96	54.68 ± 10.86	-1.50	0.135	-8.03~0.26
Female (%)(*x̄* ± *s*)	86(63.24)	24(48.00)	3.51	0.061	–
BMI (kg/m^2^)(*x̄* ± *s*)	24.07 ± 4.00	25.32 ± 3.88	-1.89	0.060	-2.52~0.02
SMI (kg/m^2^)(*x̄* ± *s*)	6.32 ± 1.22	7.20 ± 1.10	-4.47	**<0.001**	-1.25~-0.51
SMM (kg)(*x̄* ± *s*)	40.74 ± 10.39	44.96 ± 8.74	-2.54	**0.012**	-7.21~-1.23
Hand grip (kg)(*x̄* ± *s*)	34.30(18.4)	39.25 ± 12.15	-1.05	0.296	–
Cardiac measurements					
LA (mm)(*x̄* ± *s*)	31.62 ± 3.33	31.60 ± 2.97	0.03	0.974	-0.98~1.01
AO (mm)(*x̄* ± *s*)	20.79 ± 1.71	20.94 ± 1.84	-0.53	0.598	-0.74~0.43
LVDd (mm)(*x̄* ± *s*)	45.77 ± 2.92	45.60 ± 3.07	0.35	0.727	-0.81~1.15
IVSTd (mm)(*x̄* ± *s*)	8.62 ± 0.79	8.84 ± 0.92	-1.57	0.119	-0.50~0.07
LVPWTd (mm)(*x̄* ± *s*)	8.45 ± 0.69	8.70 ± 0.96	-1.95	0.053	-0.54~0.04
RV (mm)(*x̄* ± *s*)	20.91 ± 1.40	19.80 ± 3.02	3.40	**<0.001**	0.24~1.98
AAO (mm)(*x̄* ± *s*)	29.38 ± 3.06	32.04 ± 3.14	-5.19	**<0.001**	-3.67~-1.65
MPA (mm)(*x̄* ± *s*)	21.10 ± 1.33	20.50 ± 1.39	2.66	**0.009**	0.15~1.04
EF (%)(*x̄* ± *s*)	58.40 ± 2.74	60.38 ± 4.45	-3.61	**<0.001**	-3.29~-0.66
FS (%)(*x̄* ± *s*)	30.07 ± 1.69	31.54 ± 1.80	-5.13	**<0.001**	-2.04~-0.89
LVM (g)(*x̄* ± *s*)	118.58(28.12)	132.43 ± 25.52	-2.52	**0.011**	–

SMI, Skeletal Muscle Index; SMM, Skeletal Muscle Mass; BMI, Body Mass Index; LA, Left Atrial diameter; AO, Aortic Root diameter; LVDd, Left Ventricular End-Diastolic Diameter; IVSTd, Interventricular Septal Thickness at End-Diastole; LVPWTd, Left Ventricular Posterior Wall Thickness at End-Diastole; RV, Right Ventricular diameter; AAO, Ascending Aortic Diameter; MPA, Main Pulmonary Artery diameter; EF, Ejection Fraction; FS, Fractional Shortening; LVM, Left Ventricular Mass. The bold values is statistically significant (P<0.05).

#### 3.1.2 Comparison of variables between the two subgroups

Regarding clinical covariates, Group A had a significantly higher mean age compared to Group B, and there were significant differences between the two groups in terms of the proportions of male/female, smokers, and drinkers. In terms of skeletal muscle and body composition variables, Group A had significantly higher values for SMI, SMM, and grip strength compared to Group B (*P*<0.05). In terms of echocardiography results, Group A had significantly higher values for LA, AO, LVDd, IVSTd, LVPWTd, RV, AAO, MPA, and LVM compared to Group B (*P*<0.05) (see **Table 2**).

**Table 2:** The comparison of variables between two groups.

Variable	Group A (n=68)	Group B (n=68)	*t*/*z*/*x* ^2^	*P*	95%CI
Clinical covariates					
Age (years)(*x̄* ± *s*)	54.93 ± 15.91	46.66 ± 16.97	2.91	**0.004**	2.74~13.79
Female (%)(*x̄* ± *s*)	27(39.71)	59(86.76)	32.39	**<0.001**	–
BMI (kg/m^2^)(*x̄* ± *s*)	24.68 ± 4.31	23.45 ± 3.57	1.80	0.074	-0.10~2.56
Heart rate(*x̄* ± *s*)	86.81 ± 12.07	89.59 ± 12.99	-1.28	0.202	-6.99~1.44
Respiratory rate(*x̄* ± *s*)	18.87 ± 1.63	18.90 ± 1.64	-0.10	0.917	-0.58~0.52
Hypertension (%)	14(20.59)	12(17.65)	0.19	0.663	–
Dyslipidemia (%)	15(22.06)	13(19.12)	0.39	0.530	–
Smokers (%)	25(36.76)	6(8.82)	15.08	**<0.001**	–
Drankers (%)	25(36.76)	8(11.76)	11.56	**0.001**	–
FT3 (pmol/L)(*x̄* ± *s*)	16.96(11.97)	21.11(15.11)	-1.54	0.124	–
FT4 (pmol/L)(*x̄* ± *s*)	39.33(36.99)	49.07(38.00)	-1.48	0.138	–
TSH (mIU/L)(*x̄* ± *s*)	0.01(0.01)	0.01(0.01)	0.42	0.677	–
IL-6(pg/mL)	11.75(13.82)	10.81(11.14)	0.84	0.403	–
IL-15(pg/mL)	13.63(10.29)	15.55(10.60)	-1.06	0.288	–
TNF-α(pg/mL)	17.17(14.75)	20.28(17.58)	-0.94	0.348	–
Muscle variables					
SMI (kg/m^2^)(*x̄* ± *s*)	6.69 ± 1.37	5.96 ± 0.90	3.64	**<0.001**	0.34~1.12
SMM (kg)(*x̄* ± *s*)	44.46 ± 11.94	37.03 ± 6.77	4.43	**<0.001**	4.17~10.69
Hand grip (kg)(*x̄* ± *s*)	40.66 ± 12.50	33.02 ± 10.49	3.83	**<0.001**	3.76~11.52
No-sarcopenia/pre-sarcopenia/sarcopenia	42/21/5	44/18/6	0.37	0.832	
Cardiac measurements					
LA (mm)(*x̄* ± *s*)	33.13 ± 3.35	30.10 ± 2.52	5.91	**<0.001**	2.03~4.03
AO (mm)(*x̄* ± *s*)	21.12 ± 1.36	20.46 ± 1.94	2.29	**0.024**	0.10~1.23
LVDd (mm)(*x̄* ± *s*)	47.38 ± 2.78	44.16 ± 2.02	7.67	**<0.001**	2.40~4.04
IVSTd (mm)(*x̄* ± *s*)	9.13 ± 0.68	8.12 ± 0.50	9.80	**<0.001**	0.81~1.22
LVPWTd (mm)(*x̄* ± *s*)	8.94 ± 0.57	7.96 ± 0.40	11.64	**<0.001**	0.82~1.15
RV (mm)(*x̄* ± *s*)	21.25 ± 1.37	20.57 ± 1.34	2.89	**0.005**	0.22~1.13
AAO (mm)(*x̄* ± *s*)	30.69 ± 3.18	28.07 ± 2.28	5.48	**<0.001**	1.69~3.55
MPA (mm)(*x̄* ± *s*)	21.43 ± 1.34	20.76 ± 1.24	2.97	**0.004**	0.23~1.10
EF (%)(*x̄* ± *s*)	57.96 ± 2.39	58.85 ± 2.99	-1.92	0.057	-1.81~0.01
FS (%)(*x̄* ± *s*)	29.82 ± 1.74	30.32 ± 1.59	-1.73	0.085	-1.06~0.06
LVM (g)(*x̄* ± *s*)	145.88 ± 18.44	110.89 ± 8.97	13.97	**<0.001**	30.12~39.87

FT3, Free Triiodothyronine; FT4, Free Thyroxine; TSH, Thyroid-stimulating Hormone; SMI, Skeletal Muscle Index; SMM, Skeletal Muscle Mass; BMI, Body Mass Index; LA, Left Atrial diameter; AO, Aortic Root diameter; LVDd, Left Ventricular End-Diastolic Diameter; IVSTd, Interventricular Septal Thickness at End-Diastole; LVPWTd, Left Ventricular Posterior Wall Thickness at End-Diastole; RV, Right Ventricular diameter; AAO, Ascending Aortic Diameter; MPA, Main Pulmonary Artery diameter; EF, Ejection Fraction; FS, Fractional Shortening; LVM, Left Ventricular Mass; IL-6, Interleukin-6; IL-15, Interleukin-15; TNF-α,Tumor necrosis factor alpha. The bold values is statistically significant (P<0.05).

### 3.2 Multiple linear regression results for skeletal muscle parameters and LVM

We performed multiple linear regression analysis with LVM as the dependent variable and SMI, SMM, and grip strength as independent variables. We included age, gender (0=male, 1=female), Smoker (0=NO, 1=Yes), Drinker (0=NO, 1=Yes), thyroid-related hormones, IL-6, IL-15, TNF-α, dyslipidemia (0=NO, 1=Yes), and hypertension (0=NO, 1=Yes) as covariates to control for the influence of other relevant factors on LVM. The results of multiple linear regression showed that SMI and grip strength had a certain correlation with LVM (see **Table 3**), while SMI did not show a significant correlation with LVM (*β*=-3.98, *P*=0.097, 95*% CI*=-8.70-0.73). The model’s R^2 = ^0.387, indicating that it can explain approximately 38.70% of the variance in the dependent variable (LVM). Furthermore, the AIC (Akaike Information Criterion) value of 1203 and the BIC (Bayesian Information Criterion) value of 1252 suggest that the model’s fit and/or complexity may be suboptimal. The results of multiple regression indicated that the residuals did not follow a normal distribution (K-S=2.50, *P*<0.01), indicating that the assumptions of the model were not met and the distribution of the error term was not constant, which may lead to bias in the model’s predictive results (see **Table 3**).

**Table 3:** Robust regression results of skeletal muscle and body mass variables and LVM.

Variable	β	Standard Error	*t*	*P*	95%CI
Const	85.56	17.08	5.01	**<0.001**	51.74~119.38
Age	0.42	0.11	3.88	**<0.001**	0.20~0.63
Gender	-14.60	5.68	-2.57	**0.011**	-25.84~-3.36
SMM	0.60	0.29	2.06	**0.042**	0.02~1.17
Hand grip	0.34	0.17	2.02	**0.045**	0.01~0.67

SMM, Skeletal Muscle Mass. The bold values is statistically significant (P<0.05).

### 3.3 GLM results for skeletal muscle parameters and LVM

Due to the non-normal distribution of residuals in the multiple linear regression, a linear model may not capture the true relationship between skeletal muscle parameters and LVM. In order to gain a deeper understanding of the relationship between skeletal muscle and LVM, while controlling for the influence of covariates on LVM, we employed a GLM to address this issue. GLM is a flexible statistical model that allows for the extension of linear relationships to nonlinear relationships and handles data with different distributions and link functions. The results of the generalized linear model showed that SMM and grip strength had a significant impact on LVM. The model’s R^2 = ^0.430, indicating that it can explain approximately 43.00% of the variance in the dependent variable (LVM). The Log-Likelihood is -584.46. The results of multiple regression indicated that the residuals did not follow a normal distribution (K-S=0.48, *P*<0.01), indicating that the assumptions of the model were not met and the distribution of the error term was not constant, which may lead to bias in the model’s predictive results (see **Table 4**).

**Table 4:** GLM analysis results of skeletal muscle and LVM.

Variable	β	Standard Error	*z*	*P*	95%CI
const	85.56	17.08	5.01	**<0.001**	52.09~119.04
Age	0.42	0.11	3.88	**<0.001**	0.21~0.63
Gender	-14.60	5.68	-2.57	**0.010**	-25.72~-3.48
SMM	0.60	0.29	2.06	**0.040**	0.03~1.17
Hand grip	0.34	0.17	2.02	**0.043**	0.01~0.67
IL-6	0.54	0.28	1.97	**0.049**	0.002~1.081

SMM, Skeletal Muscle Mass; IL-6, Interleukin-6. The bold values is statistically significant (P<0.05).

### 3.4 SEM results for skeletal muscle parameters and LVM

Considering that the GLM model may overlook the influence of latent variables and measurement errors, as well as complex interactions among variables, we further conducted an analysis using SEM models to explore and validate the causal relationships between variables. The SEM models take into account the presence of latent variables and allow for the assessment of the robustness and consistency of the results obtained from the GLM model. The results of the SEM model still showed that SMM and grip strength had a significant impact on LVM. The model’s R^2 = ^0.387, indicating that it can explain approximately 38.70% of the variance in the dependent variable (LVM), and *F*=4.70, *P*<0.01, the model is statistically significant (see **Table 5**).

**Table 5:** SEM analysis results of skeletal muscle and LVM.

Variable	β	Standard Error	*z*	*P*	95%CI
const	85.56	17.08	5.01	**<0.001**	51.74~119.38
Age	0.42	0.11	3.88	**<0.001**	0.20~0.63
Gender	-14.60	5.68	-2.57	**0.011**	-25.84~-3.36
SMM	0.60	0.29	2.06	**0.042**	0.02~1.17
Hand grip	0.34	0.17	2.02	**0.045**	0.01~0.67

SMM, Skeletal Muscle Mass. The bold values is statistically significant (P<0.05).

**Figure 2:**
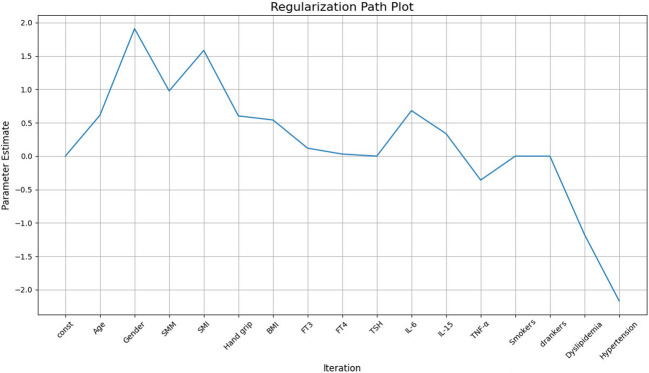
Regularized SEM diagram of the relationship between LVM and skeletal muscle.

To address the potential problem of overfitting due to the relatively small sample size (136 cases), we applied regularization techniques to constrain the structural equation model. The purpose of regularization is to reduce model complexity, avoiding excessive reliance on sample-specific noise or outliers, thus improving model stability and interpretability. After regularization, the results showed that SMM had a significant positive effect on LVM (*β*=0.96), indicating that as skeletal muscle mass increases, LVM also increases correspondingly. Grip strength (Hand grip) also had a positive effect on LVM (*β*=0.3911), suggesting that an increase in grip strength is associated with an increase in LVM (see **Figure 2**).

### 3.5 LOWESS Results for Skeletal Muscle Parameters and LVM

**Figure 3:**
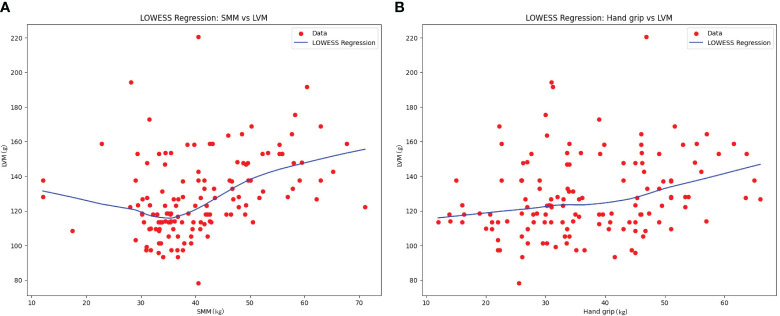
LOWESS results of the relationship between SMM, hand grip, and LVM.

To further explore the general trend between LVM and SMM, as well as between LVM and grip strength (Hand grip), LOWESS fitting was conducted. The results revealed a nonlinear trend with an initial decrease followed by an increase between SMM and LVM. There was an approximate linear increasing trend between SMM and grip strength (See **Figure 3**).

## 4 Discussion

With the increasing understanding of the relationship between cardiovascular diseases and sarcopenia in the elderly, it has been discovered that skeletal muscle loss may be associated with cardiac aging and remodeling, leading to the concept of “Cardio-Sarcopenia” as an upstream change in cardiovascular diseases (4). However, studying secondary sarcopenia is also important as it helps elucidate the underlying cardiac pathology caused by these diseases. Therefore, this study shifted its focus to the population with hyperthyroidism, which primarily affects middle-aged and young individuals and is characterized by severe muscle loss. This contributes to understanding the interaction between skeletal muscle and the heart in a younger population and aids in the prevention and management of cardiac pathology in patients with hyperthyroidism. The results of this study showed that the the Study group B had lower SMI, SMM, and LVM, low LVM may be accompanied by a decrease in skeletal muscle mass, indicating a potential association between low LVM and SMI in hyperthyroid patients. Similar findings have been reported by Kazemi-Bajestani et al. (10) in patients with liver cirrhosis, where the loss of LVM and skeletal muscle mass occurred simultaneously. Kazemi-Bajestani et al. (11) also found simultaneous atrophy of LVM and skeletal muscle loss in patients with non-small cell lung cancer. Although the pathological processes and system involvement differ among these diseases, they can all lead to metabolic disturbances, inflammatory responses, and tissue damage, resulting in a reduction in skeletal muscle mass and cardiac remodeling (12–14). These research findings further support the results of this study, suggesting that the decrease in skeletal muscle mass appears to occur concurrently with the increase in LVM in patients with chronic diseases. Chronic diseases such as hyperthyroidism, liver cirrhosis, and cancer may share some common mechanisms and influencing factors, indicating that changes in skeletal muscle mass may play a role in cardiac remodeling and are associated with changes in LVM.

Considering the potential influence of age and gender differences between the two subgroups on LVM and skeletal muscle mass, further investigations were conducted. Through the analyses of multiple linear regression, generalized linear models, and SEM models, we found correlations between age, gender, SMM, and grip strength with LVM. These findings are consistent with previous research on the elderly population (4–7), indicating a correlation between LVM and SMM and grip strength. The LOWESS regression model revealed an increasing trend and an approximate linear relationship between SMM and grip strength. In terms of the relationship between SMM and LVM, a nonlinear trend with an initial decrease followed by an increase was observed. This suggests that within a lower range of SMM, LVM gradually decreases as SMM increases, and then begins to increase at a certain point. In patients with hyperthyroidism, long-term hyperthyroidism causes remodeling of the heart, including cardiomyocyte enlargement and fibrous deposition. These changes can lead to ventricular dilation and dilated cardiomyopathy. Previous studies have confirmed the association between long-term hyperthyroidism and increased left ventricular fibrosis, diastolic dysfunction, and systolic dysfunction (15, 16). Marcisz et al. (17). found and suggests that the changes in LVM caused by hyperthyroidism are primarily due to eccentric remodeling, possibly due to volume overload and related to the hemodynamic load on the heart. Changes in cardiac blood flow caused by hyperthyroidism are influenced by multiple factors. Firstly, hyperthyroidism leads to increased contractility and ejection fraction of the heart, improved diastolic relaxation, and a significantly increased cardiac output. Secondly, hyperthyroidism causes peripheral vasodilation, reducing peripheral vascular resistance, further enhancing the cardiac pumping capacity, and keeping the heart in a high cardiac output state, even at rest, as it operates at maximum capacity (18). Skeletal muscles play an important role in reducing the cardiac workload. The muscles in the lower limbs, for example, help propel deoxygenated blood towards the heart and assist in the dilation and filling of capillaries, thereby enhancing cardiac pumping capacity. Therefore, sarcopenia and muscle weakness caused by hyperthyroidism may increase the workload on the heart and worsen the condition of cardiovascular diseases (19). Additionally, another study has shown that treatment methods involving electrical stimulation of the lower limbs to induce muscle contractions can effectively improve flow-mediated dilation in patients with chronic heart failure (20). Therefore, we propose that muscle loss caused by hyperthyroidism may have an impact on cardiac blood flow and load, exacerbating the condition of cardiovascular high-flow load and subsequently affecting LVM. This could result in myocardial atrophy and hypertrophy, as the heart needs to adapt and compensate for lower muscle mass in order to maintain its function. When muscle mass decreases to a certain extent, it may trigger adaptive mechanisms in the heart, leading to myocardial remodeling and hypertrophy.

The reduction of skeletal muscle mass and myocardium may be influenced by common mechanisms, leading to systemic protein metabolism disturbances and a decrease in skeletal muscle mass and LVM. In patients with hyperthyroidism, abnormal levels of thyroid hormones and TSH can directly affect muscle protein synthesis, resulting in a decrease in skeletal muscle mass (21). Elevated thyroid hormone levels can enhance myocardial contractility and the heart’s ability to adapt to workload. However, abnormal thyroid hormone levels may contribute to cardiomyocyte apoptosis, fibrosis, and cardiac hypertrophy (22–25). Oxidative stress and chronic inflammation associated with hyperthyroidism may also play a role in the decreased skeletal muscle mass and LVM (26). Oxidative stress can induce cardiac tissue remodeling and restructuring through the activation of autophagy, cell apoptosis, inflammation, and other mechanisms. It can also promote muscle protein degradation, cellular apoptosis, and metabolic dysfunction, leading to a decrease in muscle mass (27–29). Chronic inflammation is significant in both sarcopenia and cardiac remodeling. Inflammatory cytokines such as TNF-α and IL-6 are excessively produced and released with aging or in disease states. These inflammatory factors can disrupt skeletal muscle protein synthesis, directly participate in skeletal muscle protein degradation, and regulate compensatory mechanisms of the heart, thereby influencing the function and structure of cardiomyocytes and leading to cardiac remodeling (30–34). Oxidative stress and inflammation interact with each other, forming a vicious cycle. In an inflammatory state, excessive reactive oxygen species are released, while oxidative stress can promote the occurrence and maintenance of the inflammatory response. The interaction between oxidative stress and inflammation may have detrimental effects on the structure and function of both skeletal muscle and myocardium. In summary, the relationship between LVM and skeletal muscle may be influenced by factors such as abnormal thyroid hormone levels, oxidative stress, and chronic inflammation, which can affect the self-regulatory mechanisms of the heart and lead to abnormal hypertrophy of LVM, myocardial atrophy, or other changes in cardiac structure and function. However, the causal relationship between skeletal muscle loss and myocardial hypertrophy is still not fully understood. Further studies are needed to better understand the relationship between skeletal muscle and myocardium and their impact on cardiac health.

There are several limitations to consider in this study. Firstly, the difficulties in collecting and the limited selection criteria for patients with hyperthyroidism led to a small sample size, which may have limited the reliability and generalizability of the results. Expanding the sample size would be beneficial in future research to increase statistical power. Secondly, the retrospective analysis design used in this study may introduce information bias and systematic errors. Prospective studies with a well-designed protocol can provide more robust evidence. Furthermore, this study focused on specific factors and did not account for potential confounding variables that could influence the interpretation of the results. Future research should consider including a more comprehensive range of factors, such as nutritional status and neural regulation, to provide a more in-depth understanding of the relationship between skeletal muscle and LVM. In summary, future studies should aim for larger sample sizes, more rigorous study designs, and comprehensive considerations of potential influencing factors. Prospective studies are recommended to further investigate and clarify the relationship between skeletal muscle and LVM. Additionally, the inclusion of other relevant factors will help provide a more comprehensive understanding of this relationship.

## 5 Conclusion

This study identified simultaneous decreases in LVM, SMM and SMI in patients with hyperthyroidism. The results also indicated a correlation between LVM and SMM, as well as hand grip strength in these patients. These findings suggest an association between cardiac muscle mass, skeletal muscle mass, and hand grip strength in patients with hyperthyroidism. Further investigation into the causal relationship and underlying mechanisms between skeletal muscle and cardiac muscle, as well as interventions to improve this relationship, will contribute to a better understanding of muscle dysfunction and cardiac health issues in patients with hyperthyroidism, providing valuable insights for clinical treatment.

## Data availability statement

The availability of datasets generated from clinical data is subject to the policies and regulations of the relevant institutions involved. As a result, access to these datasets may require the approval and permission of the corresponding institutions. It is important to ensure the protection of participants’ identifiable data and only share raw, anonymized data, following the applicable regulations and ethical guidelines. Requests to access the datasets should be directed to jishaozheng2021@126.com.

## Ethics statement

This study is a retrospective study aimed at analyzing anonymized participant data to obtain scientific results related to a specific field. During the research process, strict anonymization measures were implemented by the research team to protect participant privacy and ensure the security of personal information. Directly identifiable personal information has been removed to ensure that participant identities cannot be recognized. This study was approved by the Medical Ethics Committee of the 11th Clinical College of Qingdao University (Ethics Review Number: YX30068). Written informed consent for participation was not required from the participants or the participants’ legal guardians/next of kin in accordancewith the nationallegislation and institutional requirements.

## Author contributions

ZL: Conceptualization, Data curation, Formal analysis, Methodology, Software, Writing – original draft. GL: Investigation, Methodology, Writing – original draft. YW: Investigation, Writing – original draft. CZ: Methodology, Writing – original draft. YG: Investigation, Project administration, Supervision, Writing – review & editing.
